# Feeding of the probiotic bacterium *Enterococcus faecium* NCIMB 10415 differentially affects shedding of enteric viruses in pigs

**DOI:** 10.1186/1297-9716-43-58

**Published:** 2012-07-27

**Authors:** Susanne Kreuzer, Patrycja Machnowska, Jens Aßmus, Matthias Sieber, Robert Pieper, Michael FG Schmidt, Gudrun A Brockmann, Lydia Scharek-Tedin, Reimar Johne

**Affiliations:** 1 Breeding Biology and Molecular Genetics, Humboldt-Universität zu Berlin, Invalidenstraße 42, D-10115, Berlin, Germany; 2 Federal Institute for Risk Assessment, Max-Dohrn-Str. 8-10, 10589, Berlin, Germany; 3 Veterinary Virology, Freie Universität, Philippstraße 13, D-10115, Berlin, Germany; 4 Animal Nutrition, Freie Universität Berlin, Königin-Luise-Str. 49, D-14195, Berlin, Germany; 5 Veterinary Immunology and Molecular Biology, Freie Universität, Philippstraße 13, D-10115, Berlin, Germany

## Abstract

Effects of probiotic bacteria on viral infections have been described previously. Here, two groups of sows and their piglets were fed with or without feed supplementation of the probiotic bacterium *Enterococcus faecium* NCIMB 10415. Shedding of enteric viruses naturally occurring in these pigs was analyzed by quantitative real-time RT-PCR. No differences between the groups were recorded for hepatitis E virus, encephalomyocarditis virus and norovirus. In contrast, astrovirus was exclusively detected in the non-supplemented control group. Rotavirus was shedded later and with lower amounts in the probiotic piglet group (*p* < 0.05); rotavirus-shedding piglets gained less weight than non-infected animals (*p* < 0.05). Serum titres of anti-rotavirus IgA and IgG antibodies were higher in piglets from the control group, whereas no difference was detected between sow groups. Phenotype analysis of immune cell antigens revealed significant differences of the CD4 and CD8β (*p* < 0.05) as well as CD8α and CD25 (*p* < 0.1) T cell populations of the probiotic supplemented group compared to the non-supplemented control group. In addition, differences were evident for CD21/MHCII-positive (*p* < 0.05) and IgM-positive (*p* < 0.1) B cell populations. The results indicate that probiotic bacteria could have effects on virus shedding in naturally infected pigs, which depend on the virus type. These effects seem to be caused by immunological changes; however, the distinct mechanism of action remains to be elucidated.

## Introduction

Bacteria- and virus-induced gastrointestinal disorders are a common problem in piglets. Infections are often associated with insufficient maternal immune protection, poor hygiene conditions, environmental changes, weaning stress, and dietary changes after weaning. To decrease the risk of infectious diseases, in-feed antibiotics have been used for decades. Their ban in Europe in 2006 has increased attempts to identify alternatives such as prebiotics, probiotics, organic acids, trace elements and other feed additives. In pigs, probiotics such as *Enterococcus faecium* are commonly used. Some studies show positive effects of probiotics against microbial infections in pigs [1-4].

No effective therapy exists against intestinal virus infections of pigs. Therefore, vaccination is the most promising method to actively control disease and viral shedding. However, vaccines are only available for a few viruses, whereas a wide range of viruses could be detected in porcine feces. Some of them are closely related to human viruses and are therefore supposed to have a zoonotic potential. This includes astrovirus (AstV), encephalomyocarditis virus (EMCV), hepatitis E virus (HEV), norovirus genogroup II (NoV GGII) and group A rotavirus (rotavirus A). The infections can be associated with diarrhoea. For example rotavirus A causes acute diarrhoea in weaning and post-weaning piglets as well as in children [5]. Natural infection with hepatitis E virus is subclinical in pigs but may cause disease in humans [6,7] whereas infection with EMCV is associated with myocarditis and reproductive failure in pigs [8,9]. The clinical significance of norovirus and astrovirus infection in pigs is unclear. Some studies analyzed the effectiveness of probiotic treatment against virus-induced diarrhoea in humans. Child care infants fed with Lactobacillus reuteri or Bifidobacterium lactis had fewer and shorter episodes of diarrhoea [10]. In another study with children, the infection with rotavirus could not be prevented by the prophylactic use of probiotics; however, several strains of *Lactobacillus* turned out to significantly shorten the duration of diarrhoea [11,12]. Likewise, the diarrhoeal phase was shortened in adult humans with acute diarrhoea treated with *Enterococcus (E.) faecium* NCIMB 10415 [13].

Other studies report modulatory effects of probiotics on the intestinal mucosal immunity in piglets [14-16]. Only a few studies investigated the effects of probiotics on virus infections of pigs. For instance, piglets fed with *Bifidobacterium lactis* HN019 showed a reduced severity of naturally acquired diarrhoea during weaning [17]. This effect was associated with a lower amount of rotavirus and *E. coli* shed with the feces. Another study with gnotobiotic piglets experimentally infected with human rotavirus and colonized with *Lactobacillus acidophilus* and *Lactobacillus reuteri* showed no significantly reduced rotavirus shedding of diarrhoea [18]. The rotavirus-specific antibody and B cell response were similar for piglets treated with or without the probiotic bacteria. However, colonization with the probiotic bacteria resulted in a significantly higher titre of total serum IgM as well as intestinal IgM, IgG and IgA. No sows were included in this study. However, passively acquired maternal antibodies through feeding of colostrum play a major role in prevention of rotavirus disease in piglets [19]. Infection of the sows via contact to infected animals and feces lowers the risk of the piglets for rotavirus diarrhoea, but only during the first two weeks of life [20]. Thereafter, the risk for piglet diarrhoea rises independently of the sows’ immune status. As viral shedding by the sows continues, piglets are vulnerable during this phase, and probiotics may have a positive impact on their health status at this time-point. Studies with sows and piglets revealed that *E. faecium* NCIMB 10415 modulates the intestinal immune system [16]. However, the effects on bacterial infections were contradictive: on one hand, carry over infections of *Chlamydia* from the sows to their piglets were reduced [21] and infections with pathogenic *E. coli* were less frequent [16]. On the other hand, challenge infections with *Salmonella* Typhimurium DT104 were more severe in piglets fed with *E. faecium* than in the control group [22]. It was concluded that *E. faecium* alleviates only infections with pathogenic germs that are already established in the herd. According to this thesis, the immunity of the sows and the passive immune protection of their piglets are considered as important factors, which are affected by probiotic bacteria.

To test, whether feeding of *E. faecium* NCIMB 10415 to sows and their piglets has an impact on naturally occurring enteric viral infections, we performed a feeding trial and monitored the shedding of enteric viruses. In order to gain insight into the mechanisms of action of probiotic bacteria, changes in the intestinal mucosal immune system of the piglets as well as the systemic immune system of the sows and their piglets were investigated in more detail.

## Materials and methods

### Animals, housing and feeding

The study was approved by the local state office of occupational health and technical safety “Landesamt für Gesundheit und Soziales Berlin” (LaGeSo Reg. Nr. 0347/09).

Sixteen pregnant purebred landrace sows were randomly allocated into either control (C, *n* = 8) or probiotic treatment (*P*, *n* = 8). Sows in the probiotic group were fed a diet with 4.2 to 4.3 × 10^6^ cfu/g *Enterococcus faecium (E. faecium)*NCIMB 10415 (Cylactin®, Cerbios-Pharma SA, Lugano, Switzerland) from 28 days *ante partum* (a.p.) onwards. Among the 16 sows 12 had 10 to 13 piglets per litter and were therefore used for the experiment. In both control and *E. faecium* fed groups were six sows. All animals were kept under similar conditions but in different buildings in order to prevent probiotic cross contamination. Seven days after birth, four animals per litter (2 male, 2 female) were randomly chosen and ear tagged for later tissue sampling of one piglet of every litter at four different time points. Piglets were kept with their dams until weaning at the age of 26 ± 2 days. After weaning, piglets were kept in commercial flatdeck pens with two animals per pen until 54 days of age. From the age of 12 days on, piglets had access to a non-medicated pre-starter diet. After weaning they were fed a starter diet. The starter diets of the probiotic supplemented group contained 5.1 × 10^6^ cfu/g (prestarter) and 3.6 × 10^6^ cfu/g (starter) of *E. faecium* NCIMB 10415.

### Sampling and tissue preparation

One ear tagged piglet from each litter was euthanized for blood and tissue sampling at the age of 12 ± 1 (*n* = 6), 26 ± 1 (*n* = 6), 34 ± 1 (*n* = 7) and 54 ± 2 (*n* = 8). Therefore, at each time point six animals (3 males, 3 females) were used per feeding group. Additionally, at day 34 one piglet and at day 54 two piglets more per feeding group were sampled as described previously [23]. Blood was taken via cardiac puncture under ketamine/azaperone anesthesia (4 mg/kg; 25 mg/kg bodyweight) to isolate immune cells (see below) and to prepare a hemogram for counting of immune cells per mL microscopically. Piglets were then euthanized by intracardial injection of a lethal dose of tetracaine hydrochloride, mebezonium iodide and embutramide (T61, Intervet, Unterschleißheim, Germany). Following a midline abdominal incision, the small intestine was dissected from the large intestine at the ileo-cecal junction and both segments were dissected from the mesentery. A 2 cm long distal part of continuous Peyer’s Patch from the ileum (IL PP) and discrete Peyer’s Patches from mid jejunum (Je PP) were collected in Hank's Buffered Salt Solution (HBSS). Lymph nodes (LN) of the jejunum (Je LN) and ileum (IL LN) were collected in 15 mL Falcon tubes filled with 5 mL phosphate buffered saline containing 0.2% bovine serum albumin (PBS/BSA). Feces for virus detection were collected from each piglet at the sampling time point. In addition, feces and serum samples were taken from each sow at days 28 and 7 a.p.

### Immune cell isolation

The isolation of immune cells from blood, discrete PP and continuous PP were carried out as described previously [22]. Cells were collected after passage through either a nylon mesh or pressed with a syringe piston through a 70 μm BD Cell Strainer, and flushed with PBS/BSA, to remove the connective and fat tissues. The lymphocytes and the peripheral blood mononuclear cells (PBMC) were further purified using centrifugation in a Ficoll gradient. After final lysis of erythrocytes for 5 min on ice in Erylyse-Puffer pH 7.2 – 7.4 (Morphisto GmbH), the immune cells were washed with 10 mL of PBS/BSA and centrifuged for 15 min at 390 × *g* at 4°C.

### Flow cytometry

Combinations of monoclonal antibodies against surface antigens were used to detect following cell types: T helper cells (CD4^+^, CD25^+/−^, CD8^−/dim^), cytotoxic T cells (CD8α/β^+^, CD4^−^), and B cells (CD21^+^/MHCII^+^ or membrane-IgM^+^). Staining of purified immune cell preparations for CD4α and CD8α were performed with labeled primary antibodies in a one step-incubation as described before [24]. Other immune cell surface markers CD8β, CD25, CD21, IgM were detected using unlabelled primary antibodies (Additional file 1: Table S1) After washing, cells were incubated for 15 min with subclass specific secondary antibodies (goat anti-mouse-IgG, conjugated to Fluorescein isothiocyanate (FITC), R-Phycoerythrin (PE) or Allophycocyanin (APC) (Additional file 1: Table S1). After a second wash, cells were resuspended in 1 mL of PBS/BSA. Propidium iodide (PI) (0.5 μg/mL) was added to each sample directly prior to measurement, and 40 000 living (PI^−^) lymphocytes per sample were assayed by flow cytometry (FCM) within the lymphocyte gate corresponding to their forward light and sideward light scatter signals using a FACSCalibur flow cytometer equipped with a 488 nm argon laser and a 635 nm red diode laser (Becton Dickinson, Heidelberg, Germany).

### RNA extraction and real-time RT-PCR for virus detection

A 10% (wt/vol) faecal suspension was prepared with PBS and clarified by centrifugation at 1700 × *g* for 15 min at 4°C. Viral RNA was extracted from 140 μL of the suspension using the QIAamp viral RNA mini kit (Qiagen, Hilden, Germany) according to the manufacturer’s instructions. The RNA was eluted in 60 μL buffer AVE (Qiagen, Hilden, Germany) and stored at −80°C until use. Real-time RT-PCRs were performed in an ABI PRISM 7500 cycler (Applied Biosystems, Darmstadt, Germany) using the Quantitect Probe RT-PCR Kit (Qiagen, Hilden, Germany). The sequences of the used primers and TaqMan probes are listed in Additional file 2: Table S2. Primers and probes for the detection of HEV, rotavirus A and NoV GGII as well as cycling conditions were used as described elsewhere [25-27]. Slight modifications are indicated in Additional file 2: Table S2. A comparison of published primers and probe sequences for the detection of murine mengovirus [28] with sequence data of porcine EMCVs (GenBank) indicated that murine primers are suitable to detect porcine EMCVs. Real-time RT-PCRs were carried out in 25 μL of reaction mixture containing 600 nM of each primer, 150 nM probe and 5 μL of the extracted RNA. After reverse transcription at 55°C for 60 min, an initial denaturation step at 95°C was performed for 5 min, followed by 45 cycles of amplification with denaturation at 95°C for 15 s, annealing at 60°C for 1 min and elongation at 65°C for 1 min. In order to detect a wide range of different astroviruses, two reverse primers and two probes were designed based on multiple alignments with astrovirus sequences from database (GenBank). The respective real-time RT-PCR was carried out using 750 nM of forward primer Mon 244 [29], 750 nM of each reverse primer, 200 nM of each probe and 5 μL of the extracted RNA. The cycling conditions were: 60 min at 42°C for reverse transcription and 15 min at 95°C for initial denaturation, followed by 45 cycles of amplification with denaturation at 94°C for 30 s, annealing at 50°C for 30 s and extension at 72°C for 1 min. To determine R² value, PCR-efficiency and sensitivity for each assay, standard curves were generated using a series of ten-fold dilutions of the RNA standard (see below) ranging from 10^-1^ to 10^13^ RNA molecules per reaction. All assays turned out to be linear over a range of nine logs and have R² values of 0.99. PCR-efficiency was calculated from the slope of the standard curve and ranged from 91% (HEV) to 102% (AstV).

### Quantification of genome equivalent numbers

RNA-standards were generated by cloning of the RT-PCR products and subsequent in vitro transcription as described elsewhere [30]. Briefly, viral target regions were amplified by conventional RT-PCR using the Qiagen One Step RT-PCR kit (Qiagen, Hilden, Germany) with primers and cycling conditions as described above, but without probes. After cloning into the pCR4-TOPO vector using the TOPO TA Cloning Kit for Sequencing (Invitrogen, Karlsruhe, Germany), inserts containing a flanking T7 promotor were amplified by PCR with primers M13F and M13R (Invitrogen, Karlsruhe, Germany). Resulting PCR products were purified with the QIAquick DNA purification Kit (Qiagen, Hilden, Germany) and transcribed in vitro using the MEGAscript T7 Kit (Applied Biosystems, Darmstadt, Germany) according to the manufacturer’s instructions. After a DNase I digestion step, in vitro transcripts were purified with the High Pure RNA Isolation Kit (Roche, Mannheim, Germany) and the RNA was stored at −80°C. The concentration of RNA was photo metrically measured by NanoDrop device (Thermo Fisher Scientific, Bonn, Germany) and used to calculate the number of RNA molecules per μL. Tenfold dilutions series of these RNA standards were used in quantitative real-time RT-PCRs for determination of genome equivalent numbers.

### ELISA for rotavirus A-specific antibodies

**Figure 1:**
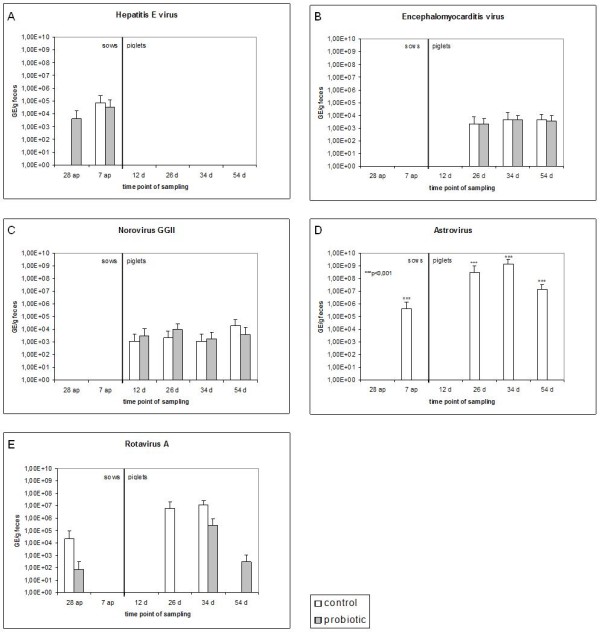
**Detection of enteric viruses in the feces of sows and their piglets using quantitative real-time RT-PCR. A)** hepatitis E virus (HEV),** B) **encephalomyocarditis virus (EMCV), C) norovirus genogroup II (NoV GGII), D) astrovirus (AstV), E) group A rotavirus (rotavirus A). White columns represent control animals, grey columns represent the probiotic supplement group. (*n* = 6/time point for the piglets; *n* = 12/time point for the sows). GE - genome equivalents per g of feces. A Wilcoxon rank-sum test was carried out. *** indicate significant differences between control and probiotic group (*p* < 0.001).

A total of 52 serum samples derived from 52 piglets at 12 d, 26 d, 34 d and 54 d and 24 serum samples at time points 28 d and 7 a.p. from 12 sows were tested for the presence of porcine anti-rotavirus A IgG and IgA antibodies using the Ingezim rotavirus porcine ELISA Kit (INGENASA, Madrid, Spain). The ELISA was performed as described in the manufacturer’s instructions. For the detection of anti-rotavirus A IgA antibodies, the ELISA was performed analogically, but the secondary antibody was exchanged by peroxidise-labelled goat anti-porcine IgA (Thermo Fisher Scientific, Bonn, Germany) at a dilution of 1/10 000. The absorbance of each sample was measured at 450 nm. The cut-off value for anti-rotavirus A IgG antibodies was derived from the manufacturer’s instructions. As it was assumed that anti-rotavirus A IgA antibodies are derived from sows only via colostrum, the cut-off value was defined on the basal values determined for sera of the piglets derived from late time-points. Therefore, for the determination of the cut-off value, the average value of the absorbance of all sera from days 34 and 54 was calculated, the standard deviation was doubled and added to the average. Normalized OD450 values were calculated by subtraction of the cut-off value from the respective OD450 value of the sample. Normalized OD450 values > 0 were considered positive and normalized OD values ≤ 0 were considered negative (negative OD450 values are not shown in Figure 1).

### Statistical analysis

Statistical analysis was performed using R 2.11.1 and SPSS 12.0.2. (SPSS, Inc., Chicago, IL, USA). All values higher than 2-times the interquartiles range below the 1^st^ quartile and above the 3^rd^ quartile were identified as outliers (less than 1% of all data). All outliers were then removed from subsequent analyses. The data were analyzed applying the general linear model and the Shapiro-Wilk test for determination of the normal distribution. Since all data were nearly normally distributed, we used raw data for the statistical tests Paired T test was performed to elucidate the effects of the feeding group. Analysis of variance (ANOVA) was performed to detect factors influencing the relative cell count of immune cells, virus shedding, and immune globulins in serum. The effects of tissue (four classes: blood, ileum lymph node, jejunum lymph node, ileal Peyers patch), age of piglets (four classes: 12, 26, 34 and 54 days of age), feeding group (two classes: feed supplemented with *E. faec*ium or not), sex (two classes) and interaction of age of piglets x feeding group were tested (nominal co-variates); sow identity was considered as a random effect. Differences were considered significant at *p* < 0.05. Box-whisker plots were chosen for graphical presentation of the results. The boxes indicate the medians (horizontal lines) and the lower and upper quartiles (lower and upper sides of the boxes). Also a Wilcoxon rank-sum test and a chi-squared test were applied to test if the numbers of shedded viruses were affected by *E. faecium*.

## Results

### Animal health status and performance

The sows did not show obvious clinical signs during the whole experiment. A few piglets from both groups had signs of diarrhoea showing liquid stool with a minor proportion of formed particles after weaning.

### Shedding of enteric viruses

**Figure 2:**
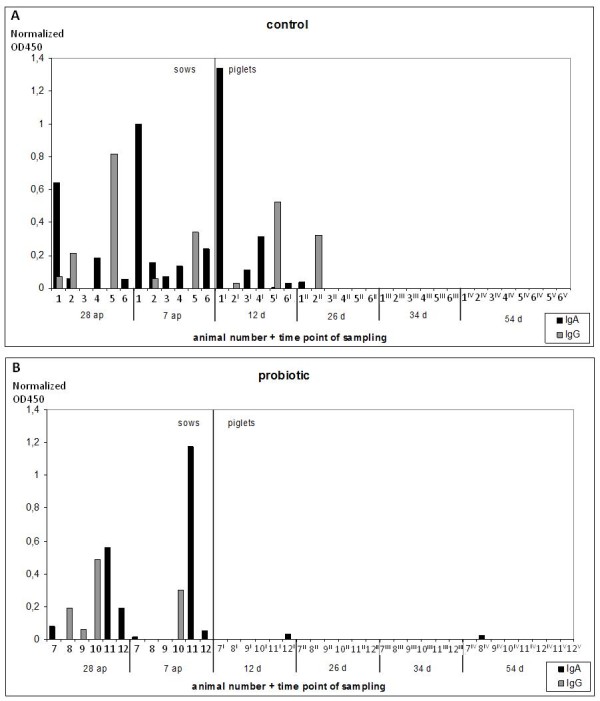
**Detection of rotavirus-specific antibodies in sows and their piglets.** Normalized OD450 values are shown for **A**) the control group and **B**) the probiotic group. Sows 1 – 6 belong to the control and sow 7 – 12 to the probiotic supplemented group. Piglets 1^x^-12^x^ represent different piglets of the sows 1–12, respectively, which were analyzed at different time-points (x). Columns are shaded grey for IgG and black for IgA.

The real-time RT-PCR tests were successfully developed for the quantitative detection of pig enteric viruses. The tests were sensitive to detect from up to 45 copies for AstV, 20 copies for EMCV, 68 copies for HEV, 78 copies for NoV GGII and 15 copies for rotavirus A. All viruses were detected in at least one of the groups (Figure 2). HEV was detected only in sows, whereas EMCV and NoV GGII were detected exclusively in piglets. No correlation was evident between virus detection and membership to the control or probiotic group for HEV, EMCV, and NoV GGII (Figure 2a-c). For AstV, nine piglets and two sows were tested positive, all animals belonging exclusively to the control group (Figure 2d). Statistical analysis indicated that the difference of astrovirus detection between the groups was highly significant (*p* < 0.001). Rotavirus A was detected in both the control and the probiotic group. At 28 days a.p., rotavirus A was detected in sows of the probiotic as well as the control group indicating that the virus was present in both groups at the beginning of the experiment. However, piglets belonging to the control group are more often infected by rotavirus A than piglets with *E. faecium* supplementation (*p* = 0.064). Altogether, 8 piglets were infected by rotavirus. In the group of infected piglets, the amount of rotavirus in the feces was two orders of magnitude lower in the probiotic group than in the control group (*p* = 0.042). Moreover, shedding of rotavirus A occurred later (*p* = 0.035) in the *E. faecium* supplemented group (Figure 2e). Analysis of the piglet growth indicates that piglets infected with rotavirus A (irrespective of *E. faecium* supplementation) gained less weight (*p* = 0.014) than the non-infected animals (Additional file 3: Figure S1).

### Humoral immunity - anti-rotavirus A IgA and IgG

Serum samples from sows and their piglets were tested for the presence of anti-rotavirus A IgG and IgA antibodies (Figure 1). Three sows of the control group and three sows of the probiotic group turned out to be positive for anti-rotavirus IgG antibodies, with decreasing titres between day 28 a.p. and day 7 a.p. Three piglets aged 12 and 26 days and belonging to the control group were tested positive for anti-rotavirus A IgG antibodies, whereas all piglets from the probiotic group turned out to be negative. Five sows of the control group and three sows of the probiotic group were positive for anti-rotavirus A IgA antibodies. In the control group, five piglets younger than 26 days where tested positive for anti-rotavirus A IgA, including one piglet with a high titre at 12 days of age. In the probiotic group, only two piglets (12 and 54 days old) were slightly positive for anti-rotavirus A IgA antibodies.

### Phenotyping of immune cell populations

**Figure 3:**
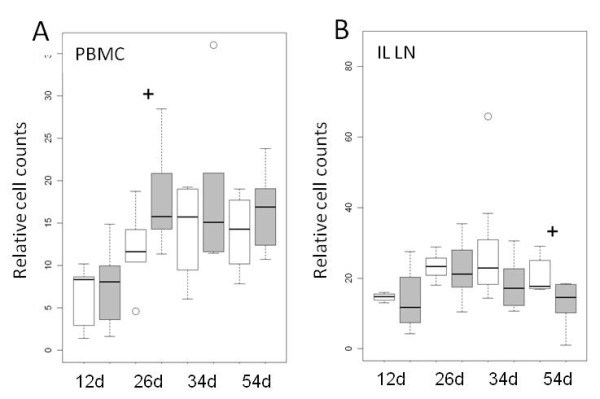
**Relative cell counts of B cells in piglets of the probiotic (*n*** **= 6 per time point, grey boxes) and the control group (*n*** **= 6 per time point, white boxes) at different days of life.** Cell counts are given as counts relative to the total number of gated living lymphocytes. **A**) B cells expressing CD21^+^MHCII^+^ in the peripheral blood mononuclear cell (PBMC) population. **B**) B cells expressing IgM on their cell surface present in ileal lymph nodes (IL LN). An ANOVA model revealed significant differences between the feeding groups (*p* = 0.043). A t-test was further performed for each age group and the results are shown (^+^0.05 < *p* < 0.1).

Immune cell populations were only phenotyped in the piglets. The flow cytometric analysis of piglet blood samples indicated, that the relative percentages of the CD21^+^/MHCII^+^ double-positive B cell population were higher in piglets of the probiotic group compared to the control group at days 26 and 54 (Figure 3a). Additional measurements of the absolute leukocyte counts/l blood and calculated absolute changes of the different immune cell population in blood, show the same tendencies for the behaviour of the immune cells for the two feeding groups (Additional file 4: Figure S2). Interestingly, the opposite of the relative distribution of B cells occurred in tissue samples from the ileum lymph nodes, with lower frequency of B cells in the control group at days 26 and 54 (Figure 3b).

**Figure 4:**
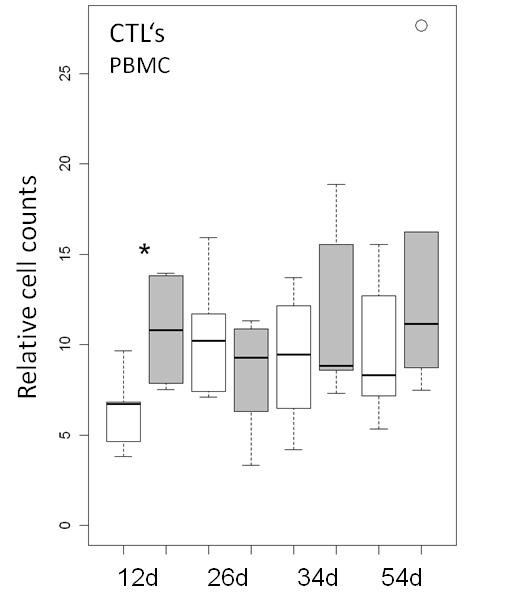
**Cell counts of CD8β**^**+**^**cytotoxic T cells (CTL’s) relative to the total number of the gated living lymphocyte population in piglets of the probiotic (*n*** **= 6 per time point, grey boxes) and the control group (n = 6 per time point, white boxes) at different days of life.** A *t*-test shows a significant difference between the feeding groups at 12 days of age (*0.01 < *p* < 0.05).

**Figure 5:**
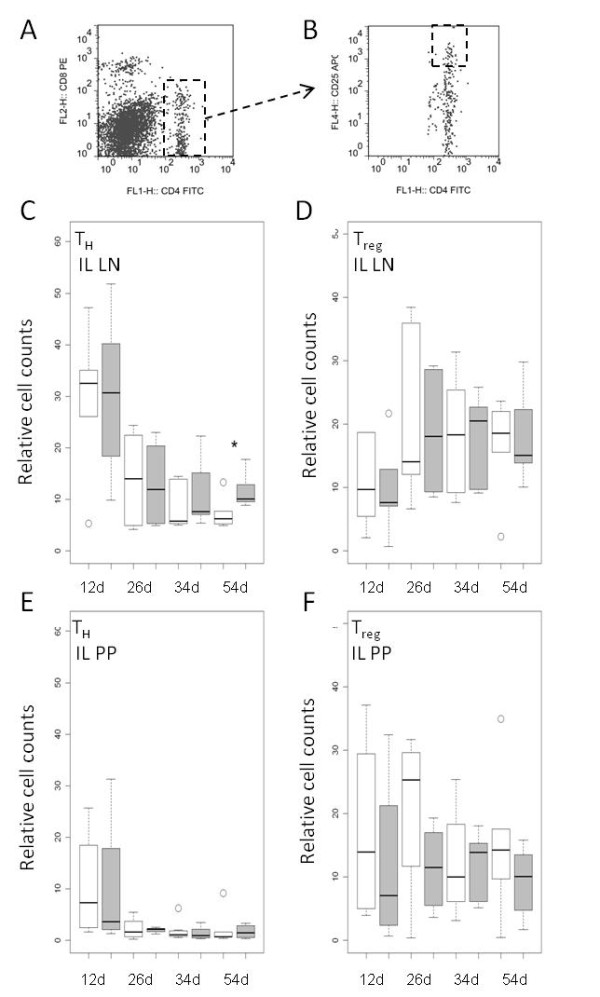
**Relative cell counts of CD4**^**+**^**CD8**^**−**^**T helper cells (T**_**H**_**) and CD4**^**+**^**CD25**^**hi**^**T regulatory helper (T**_**reg**_**) cell subpopulation in piglets of the probiotic (*n*** **= 6 per time point, grey boxes) and the control group (*n*** **= 6 per time point, white boxes) at different days of life.** Cell counts are given either for T_H_ cells as counts relative to the total number of living lymphocytes or for the T_regs_ as counts relative to the total number of T_H_ cells. **A**) and **B**) show the flow cytometry dot plots of one representative piglet. The marked population was considered as A) T_H_ and B) T_reg_ cells. **C**) T_H_ cells and **D**) T_reg_ cells in ileal lymph nodes (IL LN). **E**) T_H_ cells and **F**) T_reg_ cells in continuous ileal Peyer’s Patch (IL PP). A t-test indicated significant differences between the feeding groups in the ileal lymph nodes at 54 days of age (*0.01 < *p* < 0.05).

In the blood, CD8β^+^ cytotoxic T cells were more frequent in the probiotic group at 11 days of age (*p* < 0.05); however, this effect was not apparent around weaning time (Figure 4). CD4^+^ T helper (T_H_) cells were characterized by CD4^+^CD8α^-/dim^ gating (Figure 5a). All cells within this gate were further checked for their CD25 status and only CD4^+^CD8α^-/dim^CD25^high^ cells were considered as T regulatory (T_reg_) cells (Figure 5b). Some animals from selected time points were additionally tested for an intracellular antibody against Foxp3. We could confirm that basically all CD25high cells were also Foxp3 positive and ad versa (Additional file 5: Figure S3). Therefore only T regulatory (T_reg_) cells were included in the analysis. In the ileal lymph nodes, the relative number of CD4^+^ T_H_ cells was significantly (*p* < 0.05) increased in the probiotic group at day 54 (Figure 5c). In contrary, the percentage of T_regs_ was decreased at the same time-point (Figure 5d). In the ileal Peyer’s Patches, a comparatively high number of CD4^+^ T helper cells were detectable for both groups at 12 days of age, whereas their frequency was low at later time-points (Figure 5e). Out of these few CD4^+^ T helper cells, a considerable high percentage was identified as T_reg_ cells (Figure 5f). A more detailed description of the statistical results is presented in the Additional file 6: Tables S3 and 7: Table S4.

## Discussion

Effects of probiotic bacteria on viral infections in humans and animals have been described previously [11,12,17,18]. Most of the data from pigs are derived from experimental infection studies with pathogenic viruses [18,31,32]. However, only little is yet known about the effects on enteric viruses that occur naturally in pigs [17]. Here, two groups of sows and their piglets were fed with or without a supplementation of the probiotic bacterium *Enterococcus faecium* NCIMB 10415. By real-time RT-PCR analyses of the feces, the detection rates of enteric viruses differed between the probiotic and control group depending on the analyzed virus type. No differences between the groups were found for EMCV, HEV and NoV GGII. In contrast, astrovirus was detected only in the control group and rotavirus A was shed later and with lower amounts in the probiotic group. Although the reasons for the observed differences are not known so far, the results may indicate that probiotic bacteria are generally able to affect virus shedding in naturally infected pigs.

Recent studies have indicated that astroviruses (AstV) are highly prevalent in pigs [33,34]. But the clinical significance of AstV infection remains unclear. In our study, samples of nine piglets and two sows were tested positive for AstV, thus confirming the presence of this virus in the examined German landrace pig population. As the virus was exclusively found in the control group, an advantageous effect of the probiotic bacteria in prevention of AstV shedding may be postulated. However, it should be taken into account that the sows used in this study could only be naturally infected by AstV before the beginning of the experiment. Sows were randomly assigned to the groups and thereafter separated and kept in different buildings. Nevertheless, it cannot be totally excluded, that only animals of the control group had contact to AstV, while those of the probiotic group did not. Additional controlled experimental infection studies will be necessary to confirm the findings.

In contrast, rotavirus A was detected in both groups at the beginning of the experimental period. Therefore, it is likely that both groups had a similar exposure to rotavirus A and that the observed differences in rotavirus A shedding between the groups can be attributed to feeding with the probiotic bacterium. Positive effects of probiotics on the outcome of rotavirus disease in children have been described repeatedly [10-12,35]. Our findings in pigs are supported by published data, which indicate that an oral administration of another probiotic *Bifidobacterium lactis* HN019 could not only significantly reduce the severity of weanling diarrhoea, but also lower the concentration of rotavirus particles in the feces [17].

In order to gain insights into the possible mechanism of action of the probiotic feeding, the rotavirus A-specific humoral immune response was examined. Shu et al. [17] found increases of pathogen-specific antibody titres in feces as well as a higher response of blood leukocyte and T-lymphocyte proliferation, indicating an immune-mediated process that would be responsible for the positive effect on the rotavirus infection. Different to other studies, an increase of serum anti-rotavirus A IgA or IgG antibodies in piglets from the probiotic group was not observed in our study. In contrast, the number of antibody-positive piglets and the respective antibody titres tended to be higher in the control group. However, antibody titres in the serum may not correlate with protection against rotavirus infection; and neutralizing rotavirus-specific IgA within the gut is considered as a more important protective factor [19]. Therefore, we also analyzed the presence of B cells within lymph nodes of the gut. We found in the tissue of ileal mesenteric lymph nodes that B cells were also less frequent in the probiotic group compared to the control group. Therefore, the observed protective effect on virus shedding by supplementation of *E. faecium* seems not to be caused by an enhancement of the humoral immunity.

Besides antibody-mediated effects, other possible mechanisms of action of the probiotic bacteria may be considered as for example competitive receptor interaction [36] or clearing of infected cells by cytotoxic T cells [37]. We found especially in the youngest piglets (d12) significantly increased relative percentages of CD8β positive T cells in the probiotic group, compared to the control group (Figure 4). It is described that nearly all of the CD8β^+^ T cells belong to the CD4^+^CD8α^hi^ fraction which contains cytolytic T lymphocytes [38-40]. They recognize foreign antigens in a MHC class I-restricted manner and respond to the antigenic stimulation via proliferating, killing of the target cell, and secreting interferon γ and tumour necrosis factor α [41]. This cell population is suggested to be crucial for clearing of rotavirus A [19]. As the rotavirus A infection occurred later and less severe in the probiotic group, this observation may indicate an early immune stimulation in this group, which might be advantageous against the rotavirus A infection. By analysing CD4^+^ T helper cells, a significant increase was observed in the probiotic group only at the latest time point (day 54). As the T_reg_ cell frequency was decreased at the same time-point, this increase is probably caused by proinflammatory CD4^+^ T helper cell subtypes. This assumption has to be confirmed in future studies. As the significant CD4^+^ T helper cell increase was only detected at the end of the study, a longer monitoring time should also be scheduled in those studies.

With respect to the experimental model used in the present study, it is possible that other factors that were not taken into consideration or analyzed could have influenced the outcome. As the pigs were naturally infected, random differences regarding virus infections and specific immunity against viruses at the beginning of the experiment cannot be ruled out. The present study points out that feeding of pigs with the probiotic bacterium *Enterococcus faecium* NCIMB 10415 shows no general effects on enteric virus infections, but that excretion of specific virus types may be affected. We suggest rotavirus A and AstV as targets of *E. faecium*. A possible protective mechanism could be the early activation of cytotoxic T cells through the probiotic. Therefore, rotavirus A and AstV infections should be investigated in future studies in more controlled and longer-termed experimental animal studies as well as cell culture-based experiments to further elucidate the specific mechanisms of action.

## Competing interests

The study was funded by the Deutsche Forschungsgemeinschaft (DFG) within the Collaborative Research Group (SFB, Sonderforschungsbereich) 852 “Nutrition and intestinal microbiota - host interactions in the pig”. The authors are solely responsible for the data and do not represent any opinion of neither the DFG nor other public or commercial entity. None of the authors of this paper has a financial or personal relationship with other people or organizations that could inappropriately influence or bias the content of the paper.

## Authors’ contributions

SK carried out the sampling, detection and characterization of lymphocyte populations and drafted the manuscript. PM carried out the sampling, carried out the detection of the enteric viruses and the ELISA measurements and drafted the manuscript. JA performed statistical analysis. MS helped to carried out the sampling, detection and characterization of lymphocyte populations and contributed to writing the final version of the manuscript. RP performed the animal trial and drafted the manuscript. MFGS conceived of the study and contributed to writing the final version of the manuscript. GAB conceived of the study and contributed to the final version of the manuscript. LST carried out the sampling, detection and characterization of lymphocyte populations and drafted the manuscript. RJ conceived of the study and contributed to the final version of the manuscript. All authors read and approved the final manuscript.

## Supplementary Material

Additional file 1 Table S1. Primary and secondary antibodies (AB) used for flow cytometry staining. Click here for file

Additional file 2 Table S2. Sequences of primers and probes for real-time RT-PCR. Click here for file

Additional file 3 **Figure S1. Body weight difference between piglets infected (*n*** **=** **9) and not infected (*n*** **=** **39) with rotavirus A.** Click here for file

Additional file 4 **Figure S2. Absolute cell counts in the peripheral blood mononuclear cell (PBMC) population in piglets of the probiotic (*n*** **=** **6 per time point, grey boxes) and the control group (*n*** **=** **6 per time point, white boxes) at different days of age.** A) Absolute lymphocyte number in blood obtained by hemogram and B) absolute cell counts of B cells expressing CD21^+^MHCII^+^. Absolute cell counts are calculated from the absolute lymphocyte number in blood. Click here for file

Additional file 5 **Figure S3. Immune staining of different T cell populations in the ileal lymph nodes of one representative piglet.** The cells are within a lymphocyte gate regarding their forward sightward scatter signal and were checked for cell death by PI staining in a prior gating step. The x and y axes show the intensity of fluorescent signals of PE labeled to CD8 on the y axes and FITC labeled to CD4 on the x axes (A). Framed cells populations in A) were further analyzed in B) and the y axes show the intensity fluorescent signal of APC labeled to CD25. All lymphocytes for the same sample as shown in A) and B) for the fluorescent signals of PE were labeled to CD25 on the y axes and FITC labeled to CD4 on the x axes (C). Framed cells in C) were further analyzed in D) and the x axes show the fluorescent signal of APC labeled to the transcription factor (TF) Foxp3. Click here for file

Additional file 6 Table S3. Results of an ANOVA based on the model: #Cells ~ Tissue + age + group + sex + Tissue*age + Tissue*group + Tissue*sex. Click here for file

Additional file 7 **Table S4. Results of T tests for different cell types which reached in a former performed ANOVA a significant level.** The T test was carried out between the two feedings groups at the different sampling time points in blood (BL PBMC), ileal lymph nodes (IL LN), ileal Peyer’s Patch (IL PP) and jejunal lymph nodes (Je LN). Click here for file
