# Supplementary material for: Feeding of the probiotic bacterium Enterococcus faecium NCIMB 10415 differentially affects shedding of enteric viruses in pigs
Source: Vet Res. 2012 Jul 27;43(1):58. doi: 10.1186/1297-9716-43-58 (PMC3431279; doi:10.1186/1297-9716-43-58)
Supplement: Additional file 1 — Table S1. Primary and secondary antibodies (AB) used for flow cytometry staining. [file 1297-9716-43-58-S1.doc]

**Supplemental Table 1 Primary and secondary antibodies (AB) used for flow cytometry staining.**

| **Specificity** | **Clone** | **Isotype** | **Fluorochrome** | **Distributer** |
| --- | --- | --- | --- | --- |
| **CD25** | K231.3B2 | IgG1 | none | Acris |
| **CD4α** | 74-12-4 | IgG2b | FITC | Southern Biotech |
| **CD8α** | 76-2-11 | IgG2a | PE | Southern Biotech |
| **CD8β** | PG164A | IgG2a | none | VMRD |
| **CD21** | BB6-11C9.6 | IgG1 | none | Biozol |
| **MHCII** | MSA3 | IgG2a | none | VMRD |
| **IgM** | K52 1C3 | IgG1 | none | Biozol |
| **Secondary AB** |  |  |  |  |
| **APC-αIgG1** | pooled |  | APC | Southern Biotech |
| **PE-αIgG2a** | pooled |  | PE | Southern Biotech |
| **FITC-αIgG1** | pooled |  | FITC | Southern Biotech |
| **PE-αIgG1** | pooled |  | PE | Southern Biotech |
| **FITC-αIgG2b** | pooled |  | FITC | Southern Biotech |
