# Supplementary material for: Feeding of the probiotic bacterium Enterococcus faecium NCIMB 10415 differentially affects shedding of enteric viruses in pigs
Source: Vet Res. 2012 Jul 27;43(1):58. doi: 10.1186/1297-9716-43-58 (PMC3431279; doi:10.1186/1297-9716-43-58)
Supplement: Additional file 2 — Table S2. Sequences of primers and probes for real-time RT-PCR. [file 1297-9716-43-58-S2.doc]

**Supplemental Table 2** Sequences of primers and probes for real-time RT-PCR.

| **Virus** | **Primers and probes** | **Sequence 5`-3`** | **Reference** |
| --- | --- | --- | --- |
| **AstV** | F = Mon 244 | GGTGTCACAGGW^*^CCAAAACC | [29] |
|  | R = pAstVR  P = pAstV  P = huAstV | GAGGATGCAYGCCTCRAGCTC  FAM-caatccgtcaacgggctacagcaac-TAMRA  FAM-caatatgtcagagagcaacagcaac-TAMRA | This study |
| **EMCV** | F = Mengo 110F  R = Mengo 209R  P = Mengo 147 | GCGGGTCCTGCCGW^*^AAGT  GAAGTAACATATAGACAGACGCACAC  FAM-ATCACATTACTGGCCGAAGC-MGB | [28] |
| **HEV** | F = JVHEV-F  R = JVHEV-R  P = JVHEV-P | GGTGGTTTCTGGGGTGAC  AGGGGTTGGTTGGATGAA  FAM-TGATTCTCAGCCCTTCGC-BBQ | [25] |
| **NoV GGII** | F = QNIF2a  R = COG2R  P = QNIFS | ATGTTCAGRTGGATGAGRTTY^*^TCWGA  TCGACGCCATCTTCATTCACA  FAM-AGCACGTGGGAGGGCGATCG-TAMRA | [27] |
| **Rota-virus A** | F = NvP3-F  R = NvP3-R  P = TM-probe | accatctacacatgaccctc  ggtcacataacgcccc  FAM-ATGAGCACAATAGTTAAAAGCTAACACTGTCAA-TAMRA | [26] |

^*^base modification compared to the published sequence, W = A + T, Y = C + T; F = forward primer, R = reverse primer, P = probe.
