# Supplementary figures and images for: Feeding of the probiotic bacterium Enterococcus faecium NCIMB 10415 differentially affects shedding of enteric viruses in pigs
Source: Vet Res. 2012 Jul 27;43(1):58. doi: 10.1186/1297-9716-43-58 (PMC3431279; doi:10.1186/1297-9716-43-58)

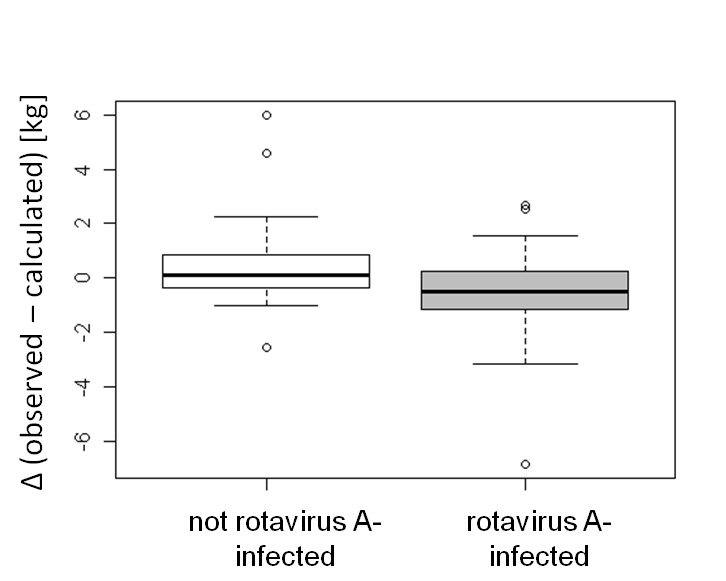

Supplement: Additional file 3 — Figure S1. Body weight difference between piglets infected (n = 9) and not infected (n = 39) with rotavirus A. [file 1297-9716-43-58-S3.tiff]

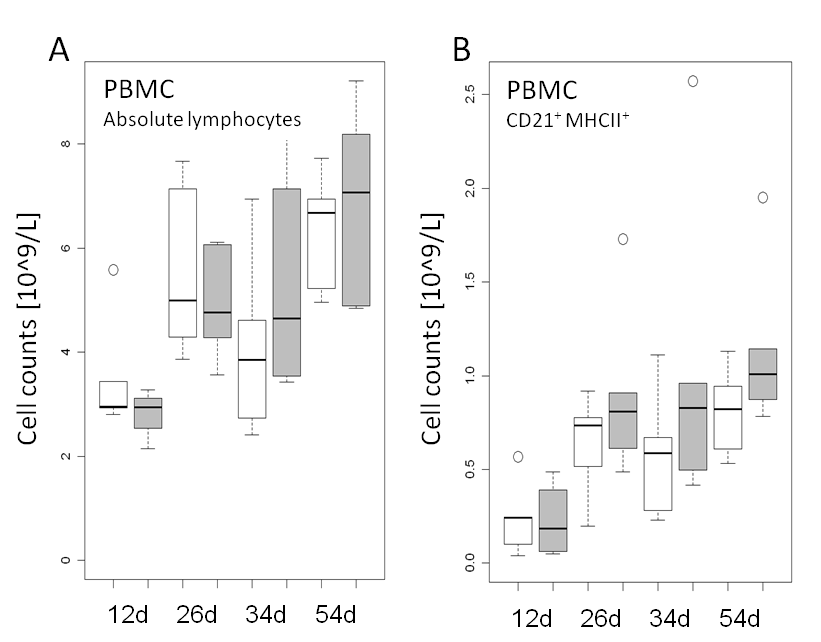

Supplement: Additional file 4 — Figure S2. Absolute cell counts in the peripheral blood mononuclear cell (PBMC) population in piglets of the probiotic (n = 6 per time point, grey boxes) and the control group (n = 6 per time point, white boxes) at different days of age. A) Absolute lymphocyte number in blood obtained by hemogram and B) absolute cell counts of B cells expressing CD21+MHCII+. Absolute cell counts are calculated from the absolute lymphocyte number in blood. [file 1297-9716-43-58-S4.tiff]

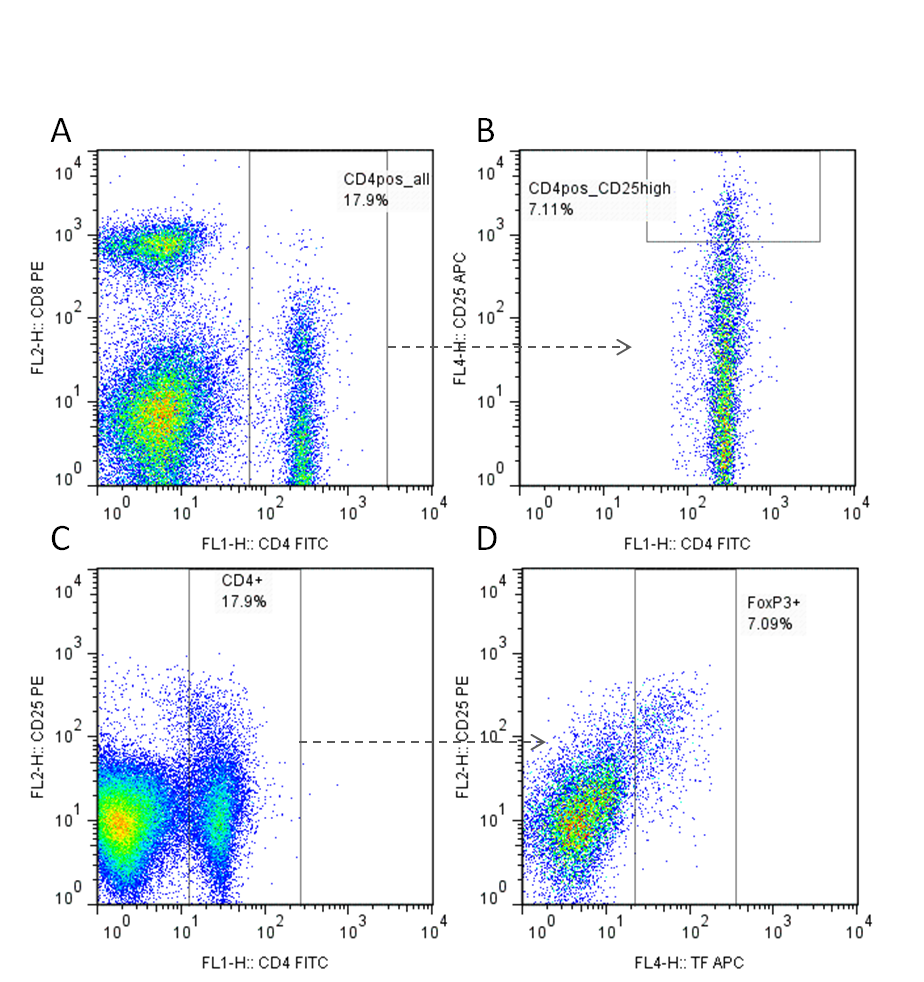

Supplement: Additional file 5 — Figure S3. Immune staining of different T cell populations in the ileal lymph nodes of one representative piglet. The cells are within a lymphocyte gate regarding their forward sightward scatter signal and were checked for cell death by PI staining in a prior gating step. The x and y axes show the intensity of fluorescent signals of PE labeled to CD8 on the y axes and FITC labeled to CD4 on the x axes (A). Framed cells populations in A) were further analyzed in B) and the y axes show the intensity fluorescent signal of APC labeled to CD25. All lymphocytes for the same sample as shown in A) and B) for the fluorescent signals of PE were labeled to CD25 on the y axes and FITC labeled to CD4 on the x axes (C). Framed cells in C) were further analyzed in D) and the x axes show the fluorescent signal of APC labeled to the transcription factor (TF) Foxp3. [file 1297-9716-43-58-S5.tiff]
