# Supplementary material for: Feeding of the probiotic bacterium Enterococcus faecium NCIMB 10415 differentially affects shedding of enteric viruses in pigs
Source: Vet Res. 2012 Jul 27;43(1):58. doi: 10.1186/1297-9716-43-58 (PMC3431279; doi:10.1186/1297-9716-43-58)
Supplement: Additional file 6 — Table S3. Results of an ANOVA based on the model: #Cells ~ Tissue + age + group + sex + Tissue*age + Tissue*group + Tissue*sex. [file 1297-9716-43-58-S6.doc]

**Supplemental Table 3 Results of an ANOVA based on the model: #Cells ~ Tissue + age + group + sex + Tissue*age + Tissue*group + Tissue*sex**

|  | **Tissue** | **SL** | **age** | **SL** | **group** | **SL** | **sex** | **SL** | **Tissue*age** | **SL** | **Tissue*group** | **SL** | **Tissue*sex** |
| --- | --- | --- | --- | --- | --- | --- | --- | --- | --- | --- | --- | --- | --- |
| **CD4^+^** | 3,98E-014 | *** | 6,79E-015 | *** | 0,906 |  | 0,276 |  | 0,001 | ** | 0,952 |  | 0,958 |
| **CD4^+^CD25^hi^** | 1,51E-007 | *** | 0,234 |  | 0,086 | + | 0,803 |  | 0,978 |  | 0,671 |  | 0,889 |
| **CD4^+^CD25^med^** | 3,46E-027 | *** | 2,52E-008 | *** | 0,126 |  | 0,469 |  | 0,189 |  | 0,285 |  | 0,772 |
| **CD4^+^CD25^-^** | 2,79E-019 | *** | 1,82E-005 | *** | 0,116 |  | 0,848 |  | 0,781 |  | 0,462 |  | 0,902 |
| **CD4^+^CD8α^dim^** | 1,54E-009 | *** | 7,44E-009 | *** | 0,911 |  | 0,538 |  | 0,147 |  | 0,915 |  | 0,910 |
| **CD4^+^CD8α^-^** | 5,74E-012 | *** | 5,70E-013 | *** | 0,986 |  | 0,364 |  | 0,001 | ** | 0,968 |  | 0,976 |
| **CD4^+^CD8α^+^** | 1,76E-007 | *** | 0,027 | * | 0,259 |  | 0,059 | + | 0,606 |  | 0,011 | * | 0,719 |
| **CD4^-^CD8α^+^** | 2,55E-014 | *** | 0,003 | ** | 0,713 |  | 0,237 |  | 0,042 | * | 0,949 |  | 0,859 |
| **CD21^+^MHCII^+^** | NA |  | 0,003 | ** | 0,043 | * | 0,649 |  | 0,832 |  | NA |  | NA |
| **CD8β^+^** | NA |  | 0,385 |  | 0,113 |  | 0,974 |  | 0,260 |  | NA |  | NA |
| **IgM^hi^** | 8,07E-011 | *** | 0,000 | *** | 0,298 |  | 0,396 |  | 0,966 |  | 0,788 |  | 0,824 |

NA: not available; SL: significant level (****p*< 0.001, ** *p* <= 0.01, **p* < 0.05, +*p* < 0.1).
