# Supplementary material for: Feeding of the probiotic bacterium Enterococcus faecium NCIMB 10415 differentially affects shedding of enteric viruses in pigs
Source: Vet Res. 2012 Jul 27;43(1):58. doi: 10.1186/1297-9716-43-58 (PMC3431279; doi:10.1186/1297-9716-43-58)
Supplement: Additional file 7 — Table S4. Results of T tests for different cell types which reached in a former performed ANOVA a significant level. The T test was carried out between the two feedings groups at the different sampling time points in blood (BL PBMC), ileal lymph nodes (IL LN), ileal Peyer’s Patch (IL PP) and jejunal lymph nodes (Je LN). [file 1297-9716-43-58-S7.doc]

**Supplemental Table 4** **Results of T tests for different cell types which reached in a former performed ANOVA a significant level.** The *T* Test was carried out between the two feedings groups at the different sampling time points in blood (BL PBMC), ileal lymph nodes (IL LN), ileal Peyer’s Patch (IL PP) and jejunal lymph nodes (Je LN).

| **Cells** | **Tissue** | **Age group** | ***t*_test** | **SL** |
| --- | --- | --- | --- | --- |
| CD4^+^ | BL PBMC | 11 | 0.763 |  |
|  |  | 26 | 0.857 |  |
|  |  | 34 | 0.785 |  |
|  |  | 56 | 0.514 |  |
|  | IL LN | 11 | 0.927 |  |
|  |  | 26 | 0.819 |  |
|  |  | 34 | 0.474 |  |
|  |  | 56 | 0.047 | * |
|  | IL PP | 11 | 0.955 |  |
|  |  | 26 | 0.865 |  |
|  |  | 34 | 0.618 |  |
|  |  | 56 | 0.730 |  |
|  | JE LN | 11 | 0.747 |  |
|  |  | 26 | 0.739 |  |
|  |  | 34 | 0.883 |  |
|  |  | 56 | 0.391 |  |
| CD21^+^MHCII^+^ | BL PBMC | 11 | 0.661 |  |
|  |  | 26 | 0.094 | + |
|  |  | 34 | 0.376 |  |
|  |  | 56 | 0.326 |  |
| CD8β | BL PBMC | 11 | 0.035 | * |
|  |  | 26 | 0.292 |  |
|  |  | 34 | 0.411 |  |
|  |  | 56 | 0.264 |  |
| IgM^hi^ | BL PBMC | 11 | 0.768 |  |
|  |  | 26 | 0.857 |  |
|  |  | 34 | 0.864 |  |
|  |  | 56 | 0.499 |  |
|  | IL LN | 11 | 0.881 |  |
|  |  | 26 | 0.792 |  |
|  |  | 34 | 0.208 |  |
|  |  | 56 | 0.052 | + |
|  | IL PP | 11 | 0.990 |  |
|  |  | 26 | 0.802 |  |
|  |  | 34 | 0.686 |  |
|  |  | 56 | 0.205 |  |
|  | JE LN | 11 | 0.276 |  |
|  |  | 26 | 0.848 |  |
|  |  | 34 | 0.926 |  |
|  |  | 56 | 0.915 |  |

SL: significance level
